# Human umbilical cord mesenchymal stem cell-derived TGFBI attenuates streptozotocin-induced type 1 diabetes mellitus by inhibiting T-cell proliferation

**DOI:** 10.1007/s13577-023-00868-9

**Published:** 2023-02-25

**Authors:** Chushan Wu, Weijiang Liu, Yuanlin Liu, Tingting Xu, Man Li, Xue Li, Yang Wang, Guangyu Meng, Lu Li, Rongxiu Zheng, Yi Zhang

**Affiliations:** 1 Department of Pediatrics, Tianjin Medical University General Hospital, 154 Anshan Road, Tianjin, 300052 People’s Republic of China; 2 Department of Experimental Hematology and Biochemistry, Beijing Institute of Radiation Medicine, 27 Taiping Road, Beijing, 100850 People’s Republic of China

**Keywords:** Mesenchymal stem or stromal cells, Type 1 diabetes mellitus, Transforming growth factor beta induced (TGFBI)

## Abstract

MSCs have been demonstrated to have a great benefit for type 1 diabetes mellitus (T1DM) due to their strong immunosuppressive and regenerative capacity. However, the comprehensive mechanism is still unclear. Our previous study indicated that transforming growth factor beta induced (TGFBI) is highly expressed in human umbilical cord-derived mesenchymal stem or stromal cells (hUC-MSCs), which are also implicated in T1DM. In this study, we found that infusion of TGFBI knockdown hUC-MSCs displayed impaired therapeutic effects in T1DM mice and decreased immunosuppressive capability. TGFBI knockdown hUC-MSCs could increase the proportion of T-cell infiltration while increasing the expression of IFN-gamma and interleukin-17A in the spleen. In addition, we also revealed that hUC-MSC-derived TGFBI could repress activated T-cell proliferation by interfering with G1/S checkpoint CyclinD2 expression. Our results demonstrate that TGFBI plays a critical role in MSC immunologic regulation. TGFBI could be a new immunoregulatory molecule controlling MSC function for new treatments of T1DM.

**Graphical Abstract:**

Schematic Representation of the Immunosuppression capacity of hUC-MSC by TGFBI

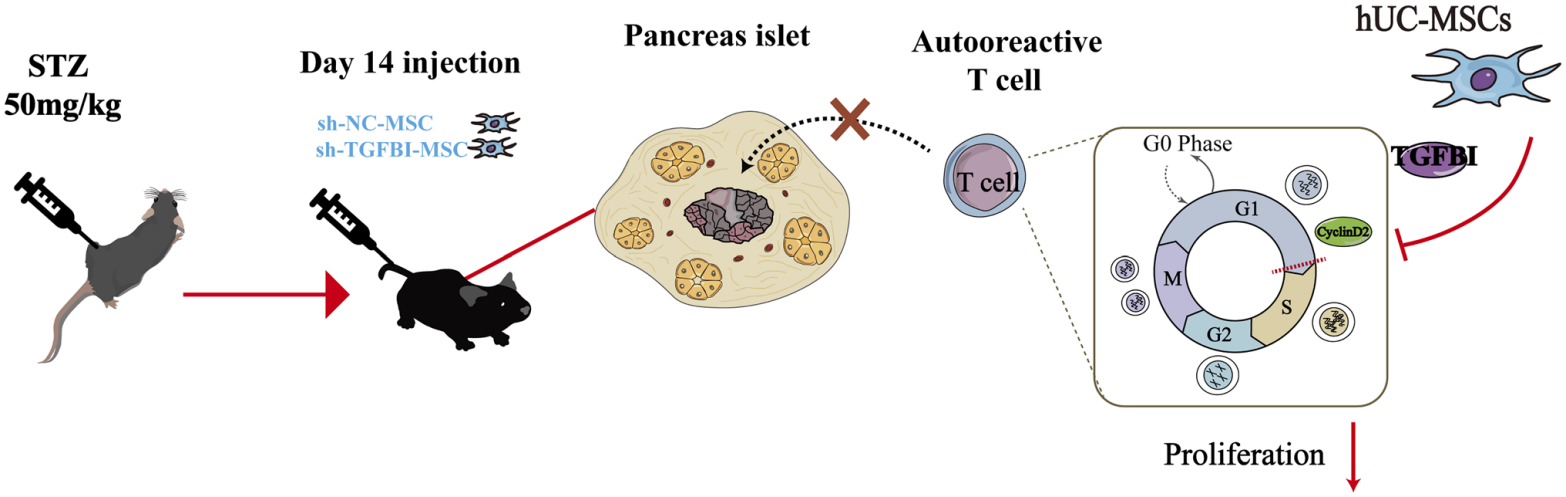

**Supplementary Information:**

The online version contains supplementary material available at 10.1007/s13577-023-00868-9.

## Introduction

Type 1 diabetes mellitus (T1DM) is a chronic autoimmune disease characterized by increased blood glucose levels (hyperglycemia), which are due to insulin deficiency that occurs as a consequence of the loss of pancreatic islet β-cells [1]. The morbidity and mortality of T1DM have increased in recent years [2]. Although hereditary, genetic, and environmental backgrounds are thought to be involved, the etiology of the disease remains unclear at present [3]. Lifelong exogenous insulin administration is the standard treatment for T1DM; however, it is difficult to maintain physiological insulin levels, and therefore, this treatment is not very effective in alleviating T1DM complications such as retinopathy, nephropathy, and neuropathy [4]. Islet transplantation is a promising strategy in type 1 diabetes mellitus therapy. However, these treatments are limited by a shortage of donor organs and the requirement for life-long immunosuppression [5].

Mesenchymal stem or stromal cells (MSCs) are multipotent progenitor cells that can migrate to sites of injury and exert immunomodulatory, tissue repair, and regenerative functions. They can be isolated from different fetal and adult tissues, such as adipose tissue, umbilical cord, bone marrow, and placenta. MSCs exert their immunosuppressive functions by secreting soluble factors and modulating the functions of various immune cells, including T lymphocytes, B cells, macrophages, and dendritic cells [6, 7]. Preclinical and clinical studies have demonstrated that MSCs can modulate immune cells, delay the onset of T1DM in mice and reduce hyperglycemia after onset with or without islet transplantation [8–10]. However, the underlying mechanisms by which MSCs alleviate immune-mediated diabetes mellitus remain unclear. In addition, different sources of mesenchymal stem/stromal cells have distinct characteristics [11, 12]. To identify the immune functional properties from different sources of MSCs, RNA sequencing was performed on MSCs isolated and cultured from umbilical cord, amniotic membrane, placenta, and bone marrow [13]. Our data showed that transforming growth factor beta-induced gene (TGFBI) was highly expressed in MSCs obtained from human umbilical cord. TGFBI, also known as βig-H3, is a gene cloned from TGFβ-stimulated A549 lung adenocarcinoma cells [14, 15]. It is a secreted protein in the ECM and has an N-terminal secretory signal, four FAS1 homologous internal domains, and a cell attachment site (RGD) at its C-terminus [16]. Previous studies revealed that three single nucleotide polymorphisms of TGFBI were significantly associated with T1DM risk and that recombinant TGFBI could preserve the integrity and enhance the function of cultured pancreatic islet cells [17, 18]. Recent studies have shown that TGFBI has an immunosuppressive function and is able to repress diabetogenic T-cell activation by interfering with early activation of the TCR signaling pathway [19]. However, its role in T1DM immunoregulatory function requires further study.

In the current study, we showed that hUC-MSCs knockdown of TGFBI reduced their ability to inhibit T-cell proliferation and impaired therapeutic effects in T1DM mice. Further mechanistic studies revealed that TGFBI regulates the immunomodulatory capacity of hUC-MSCs via repression of CyclinD2 expression. Taken together, these results reveal a novel role of TGFBI in the therapeutic effects of MSCs in T1DM mice and provide a novel mechanism for understanding the immunosuppressive capacity of MSCs.

## Materials and methods

### Cell preparation

We isolated and cultured hUC-MSCs as previously described. Human umbilical cord (hUC) specimens were obtained from normal full-term pregnancies according to the regulations of the Research Ethics Committee of Academy of Military Medical Sciences (AF/SC-08/02.255). hUC-MSCs were cultured in alpha-MEM (2357140, Gibco, Grand Island, USA) containing 10% fetal bovine serum (FBS, ST200303, PAN, Adenbach, Germany), 100 U/ml penicillin, and 100 mg/ml streptomycin and kept at 37 °C with 5% CO_2_. Upon reaching 70–80% confluency, the cells were digested with 0.125% trypsin and then cultured. Third passage cells were used for measuring phenotypes and differentiation characterization.

hUC-MSCs with TGFBI knockdown were prepared according to the instrument protocol. hUC-MSCs transfected with TGFBI-targeting shRNA carried on a lentiviral vector (GV493/hU6-MCS-CBh-gcGFP-IRES-puromycin) (GeneChem, Shanghai, China). The shRNA target sequences were as follows: TGFBI-RNAi (81): CACCACTATCCTAATGGGATT; TGFBI-RNAi (82): TGCCAAGGAACTTGCCAACAT; TGFBI-RNAi (83): GCCCTACCACTCTCAAACCTT. hUC-MSCs were incubated with lentivirus and HitransG P (REVG005, Gene Chem, Shanghai, China) for 16 h. Puromycin (2 mM) was added to the culture medium to select transduced cells.

### Differentiation assay of hUC-MSCs

To identify the multipotency of hUC-MSCs, osteogenic, adipogenic and chondrogenic differentiation assays of hUC-MSCs were performed. Cells were seeded at 2 × 10^4^ cells for osteogenic differentiation and 8 × 10^4^ cells for adipogenic differentiation in 6-well plates. The osteogenic induction medium was based on alpha-MEM containing 10% FBS with 10^− 7^ mM dexamethasone, 0.5 mM ascorbic acid, and 10 mM β-glycerol phosphate, and the adipogenic induction medium was alpha-MEM containing 10% FBS with 10^− 3^ mM dexamethasone, 0.5 mM isobutyl methylxanthine, 0.2 mM indomethacin, and 10 μg/ml insulin. The medium was changed every 2 to 3 days. After 2 weeks, the cells were washed with phosphate-buffered saline (PBS) and stained with Oil Red O to visualize the lipid droplets accumulated in the adipocytes or with alkaline phosphatase (ALP) to visualize the ALP activity in the osteoblasts. For chondrogenic differentiation, 2 × 10^5^ cells were pellet cultured in a 15 ml tube and induction medium based on alpha-MEM containing 10% FBS with 50 ng/ml TGF-β1, 50 μg/ml ascorbic acid, 1 mM sodium pyruvate, 6.25 μg/ml bovine insulin, 6.25 μg/ml transferrin, 6.25 μg/ml seleninic acid, and 1.25 μg/ml BSA. The medium was changed every 3 days. After 6 weeks, chondrogenic cell aggregate was fixed with 4% phosphate-buffered paraformaldehyde, embedded in paraffin, and sliced into 5-μm-thick sections for Alcian blue staining.

### Flow cytometry

Flow cytometric analysis was performed on the FACScalibur flow cytometer system (Beckman Coulter, Fullerton, CA, USA), and data were analyzed with FlowJo10 software (Treestar, Ashland, OR, USA). Cells were resuspended in PBS and then labeled with phycoerythrin (PE)-conjugated monoclonal antibodies against human CD73 (12-0739-42, Invitrogen, CA, USA), CD105 (12-1057-42, Invitrogen, CA, USA), and CD90 (12-0909-42, Invitrogen, CA, USA) and fluorescein isothiocyanate (APC)-conjugated antibodies against human CD34 (17-0349-42, Invitrogen, CA, USA), CD45 (17-0459-42, Invitrogen, CA, USA), and CD14 (17-0149-42, Invitrogen, CA, USA).

For intercellular cytokine staining, splenic lymphocytes were collected and then stimulated with Cell Stimulation Cocktail (TNB-4970-UL100, TONBO Biosciences, CA, USA) for 8 h. Splenic lymphocytes were stained with anti-mouse CD3-PE (12-0031-82, eBioscience, CA, USA) and anti-mouse CD4-FITC (11-0041-85, eBioscience, CA, USA) for 30 min at 4 °C. Cells were then fixed and permeabilized with Fixation/Permeabilization Diluent (TNB-1020-l050, TONBO Biosciences, CA, USA). For cytokine staining, the cells were stained with anti-mouse IFN-γ-APC (20-7311-U100, TONBO Biosciences, CA, USA) or anti-mouse IL-17-APC (17-7177-82, eBioscience, CA, USA) for 30 min at 4 °C. Signals were recorded by flow cytometry with a FACScalibur flow cytometer system (Becton Dickinson), and data were analyzed with FlowJo10 software.

### Mice

Six-week-old C57BL/6 male mice were purchased from Beijing Vital River Laboratory Animal Technology. The protocol of animal experiments was approved by the Ethics Committee of the Beijing Academy of Military Medical Sciences. The Protocol permission number is IACUC-DWZX-2021-704.

### STZ-induced murine T1DM model

All mice were randomly allocated to the control group or the diabetic group. The T1DM mouse model was induced by STZ (V900890-1G, Sigma, Darmstadt, Germany) as previously described [9, 20]. Briefly, the experimental group mice were intraperitoneally injected with STZ (50 mg/kg/d) in the lower left abdomen for 5 consecutive days. The control group mice received an equal volume of vehicle treatment. T1DM was defined as a tail blood glucose concentration ≥ 16.7 mmol/L for 3 consecutive days. After the T1DM model was established, sh-NC-MSCs (1 × 10^6^ cells), sh-TGFBI-MSCs (1 × 10^6^ cells), and PBS (equal volume) were intravenously administered to T1DM mice on days 1 and 14 (*n* = 6, three independent experiments). After treatment, the general condition of T1DM mice was monitored, and random blood glucose and body weight were measured each week.

### Quantitative real-time PCR

Total RNA was extracted with TRIzol reagent (T9424, Sigma–Aldrich, Darmstadt, Germany), followed by reverse transcription using M-MLV reverse transcriptase (2461, Takara Biomedical Technology, Japan) to cDNA. The reverse-transcribed cDNA was subjected to real-time PCR with SYBR Green reagent (CW2624, CWBIO, Beijing, China) to determine specific gene expression. The relative mRNA expression was calculated by the ΔΔCt method. The primers are listed in Supplementary Table 1.

### Western blot

Total protein from cells was extracted using RIPA lysis buffer (CW2333, CWBIO, Beijing, China) supplemented with 1 mmol/L phenylmethylsulfonylfluoride (A32961, Thermo Fisher, MA, USA), followed by separation with 10% sodium dodecyl sulfate–polyacrylamide gel electrophoresis and transfer onto PVDF membranes. The membranes were then blocked in TBST containing 5% milk for 1 h at room temperature and incubated at 4 °C overnight with rabbit anti-TGFBI (2719S, Cell Signaling Technology, MA, USA) and rabbit anti-cyclin D2 (3741, Cell Signaling Technology, MA, USA). Proteins were visualized using HRP-conjugated secondary antibodies. The bands were exposed by an ECL Kit as previously described. (17001102, Millipore, MA, USA).

### Lymphocyte proliferation assays

MSCs (5 × 10^3^ cells) were plated into 96-well plates (Corning) coated with purified anti-mouse CD3ε (2 μg/ml, 145-2C11, Biolegend, CA, USA). Splenic lymphocytes were isolated from 6-week-old C57BL/6 mice and filtered through a cell strainer (40 μm). Erythrocytes were lysed using erythrocyte lysis buffer to remove red blood cells and then centrifuged at 300 × *g* for 10 min. CD3^+^ T cells were sorted by a Pan T isolation kit (130-096-535, Miltenyi Biotec, Germany) and then resuspended in PBS. 5,6-Carboxyfluorescein diacetate succinimidyl ester (CFSE, 65-0850-84, eBioscience, CA, USA) was added at a final concentration of 2.5 μM and incubated in the dark for 10 min at 37 °C. RPMI 1640 (31800105, Gibco, Grand Island, USA) containing 10% FBS was added to stop labeling, and then the cells were centrifuged at 450 *g* for 7 min. Suspended CD3^+^ T cells with RPMI 1640 containing 10% FBS were plated into 96-well plates (MSC: CD3^+^ *T* = 1:5) in the presence or absence of MSCs. After coculture for 72 h, CD3^+^ T cells were collected and analyzed by flow cytometry.

### Hematoxylin and eosin staining

The pancreas samples were fixed with 4% phosphate-buffered paraformaldehyde, embedded in paraffin, and sliced into 4-μm-thick sections for conventional hematoxylin and eosin (H&E) staining. Next, the morphologies of the targeted organs were observed and imaged under a microscope.

### Immunohistochemistry

Dewaxed pancreatic tissue sections were immersed in antigenic repair solution at heating. The sections were washed in 3% H_2_O_2_ for 25 min to eliminate endogenous peroxidase activity and blocked with 3% BSA at room temperature. After that, the sections were incubated overnight at 4 °C with the primary antibody anti-insulin. After three washes with PBS for 10 min each time, the sections were incubated for 50 min at room temperature with the secondary antibody with HRP, and DAB was used for immunostaining. Finally, the sections were counterstained with hematoxylin, dehydrated and mounted.

### Immunofluorescence

Dewaxed pancreatic tissue sections were immersed in antigenic repair solution at heating. Subsequently, the sections were incubated with spontaneous fluorescence quenching agent for 5 min and washed with PBS for 10 min. After blocking with 3% BSA for 30 min, the samples were incubated with anti-mouse CD3 (GB111337, Servicebio, Wuhan, China) primary antibodies at 4 ℃ overnight in a wet box. The sections were incubated with secondary antibodies at room temperature for 50 min, and nuclei were stained with DAPI. Finally, the sections were observed and imaged under a fluorescence microscope.

### Cell cycle analysis

The supernatant of sh-NC-MSCs and sh-TGFBI-MSCs was ultrafiltered through a 30 kD ultrafiltration tube (UFC9030, Millipore, MA, USA). Spleen lymphocytes (1 × 10^6^ cells) were plated into 24-well plates (Corning) coated with purified anti-mouse CD3ε (2 μg/ml, 145-2C11, Biolegend, CA, USA) and cultured with RPMI 1640 medium containing 10% FBS, CD28 (1 µg/ml, 37.51, Biolegend, CA, USA), GlutaMax-I (35050061, Gibco, Grand Island, USA) and ultrafiltered MSC supernatant for 48 h. Cells in the difference group were then centrifuged at 300 × *g* for 5 min and fixed with 70% ethanol at 4 °C overnight. Fixed cells were washed and incubated in PBS supplemented with 50 μg/ml propidium iodide (PI) (P4864, Sigma–Aldrich, Darmstadt, Germany) and 100 μg/ml RNase (RNASEA-RO, Sigma–Aldrich, Darmstadt, Germany) for 30 min. Flow cytometry was used to measure DNA contents.

### Statistical analysis

Shapiro–Wilk test were used by SPSS 22.0 to analyze the distribution of data. The data conformed to normally distributed and presented as the mean ± standard error of the mean (SD). The statistical significance of the differences between two groups was analyzed by unpaired t test with SPSS 22.0 statistical software. Multiple group comparisons were used for one-way analysis of variance (ANOVA). *P* value < 0.05 was considered to be statistically significant[21, 22].

## Results

### Knockdown of the TGFBI gene in hUC-MSCs by lentiviral vector transfection

**Fig. 1:**
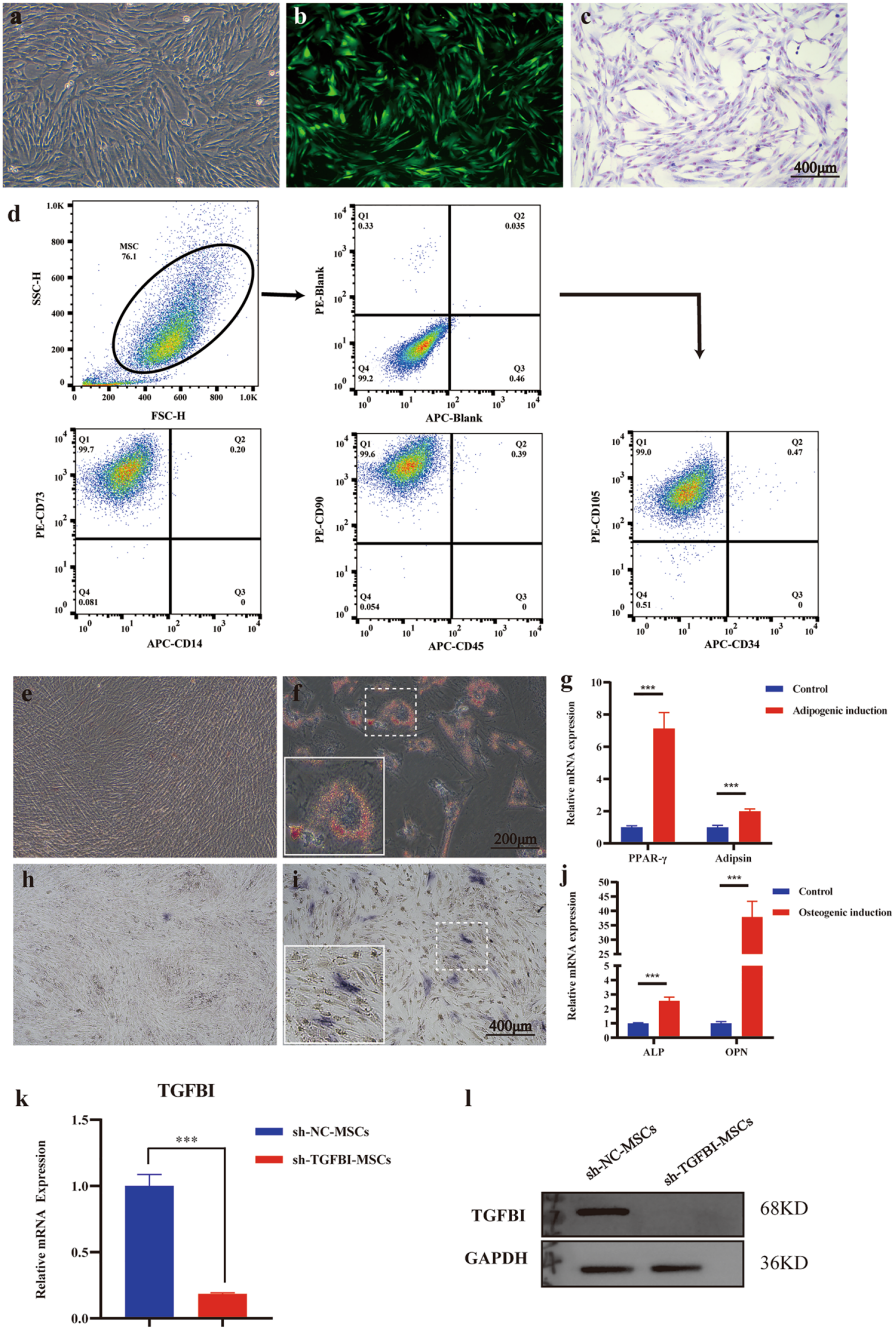
Knocking down the TGFBI gene in hUC-MSCs by lentiviral vector transfection. **a**–**c** The morphology of TGFBI knockdown hUC-MSCs (sh-TGFBI-MSCs). **a** Morphology of sh-TGFBI-MSCs. **b** Morphology of sh-TGFBI-MSCs observed by fluorescence microscopy. **c** sh-TGFBI-MSCs stained with Giemsa. **d** The cell surface markers of sh-TGFBI-MSCs were detected by flow cytometry. **e** and **f** Adipogenic differentiation of sh-TGFBI-MSCs was detected by Oil red O staining. **e** indicates the control group, and **f** indicates the adipogenic induction group. **g** PPAR-γ and adipsin mRNA expression was analyzed by qPCR. **h** and **i** Osteogenic differentiation of sh-TGFBI-MSCs was detected by alkaline phosphatase (ALP) staining; **h** indicates the control group, and **i** indicates the osteogenic induction group. **j** The mRNA levels of ALP and osteopontin (OPN) were analyzed by qPCR. **k** qPCR analysis of TGFBI knockdown efficiency in hUC-MSCs transfected with shRNA for the nontargeting sequence (sh-NC-MSCs) or shRNA for the TGFBI sequence, ***P < 0.001. **l** The protein level of TGFBI was detected by Western blot

According to our previous RNA sequencing results for different sources of MSCs, including MSCs derived from human umbilical cord, amniotic membrane, placenta, and bone marrow [13], we found that TGFBI, an extracellular matrix protein induced by TGF-β signaling, was highly expressed in human umbilical cord-derived hMSCs (hUC-MSCs) (Supplemental Fig. 1). To explore the role of TGFBI in the immune function of hUC-MSCs, we constructed a TGFBI knockdown lentivirus vector and transduced it into hUC-MSCs. This group of cells was defined as sh-TGFBI-MSCs in the following experiments. MSCs transduced with the control lentivirus were also referred to as the negative control (sh-NC-MSCs). TGFBI was successfully knocked down in sh-TGFBI-MSCs at the mRNA and protein levels, as detected by qPCR and Western blotting (Fig. 1k and l). TGFBI was continuously and stably highly expressed in different passages of hUC-MSCs under the inflammatory microenvironment and significantly higher than that of the classical immune negative regulatory molecule TGF-β1 in MSCs (Supplemental Fig. 1).

To investigate whether TGFBI knockdown MSCs still have characteristics of MSCs, we identified sh-TGFBI-MSCs from morphology, cell phonotype and induction differentiation. The results showed that sh-TGFBI-MSCs presented fibroblast-like cell morphology (Fig. 1a–c). The cell surface markers of sh-TGFBI-MSCs were positive for CD73, CD90, and CD105 but negative for CD14, CD34, and CD45 [23] (Fig. 1d).

Sequentially, we examined adipogenic and osteogenic differentiation of sh-TGFBI-MSCs in vitro. As shown in Fig. 1e and f, under adipogenic conditions, many Oil Red-O-positive lipid droplets were observed in sh-TGFBI-MSCs. Consistent with the Oil red O staining results, the mRNA expression of PPAR-γ and adipsin in the adipogenic induction group increased (Fig. 1g). Under osteogenic conditions, sh-TGFBI-MSCs displayed alkaline phosphatase (ALP) activity (Fig. 1h and i). qPCR assay results demonstrated that ALP and osteopontin (OPN) mRNA expression in the osteogenic induction group was significantly higher than that in the non-induction group (Fig. 1j). These data suggest that knocking down TGFBI does not alter the characteristics of MSCs, consistent with our previously published results [24]. In addition, sh-NC-MSCs showed similar biological characteristics (Supplemental Fig. 1).

### TGFBI deletion impaired the therapeutic efficacy of hUC-MSCs in T1DM mice

**Fig. 2:**
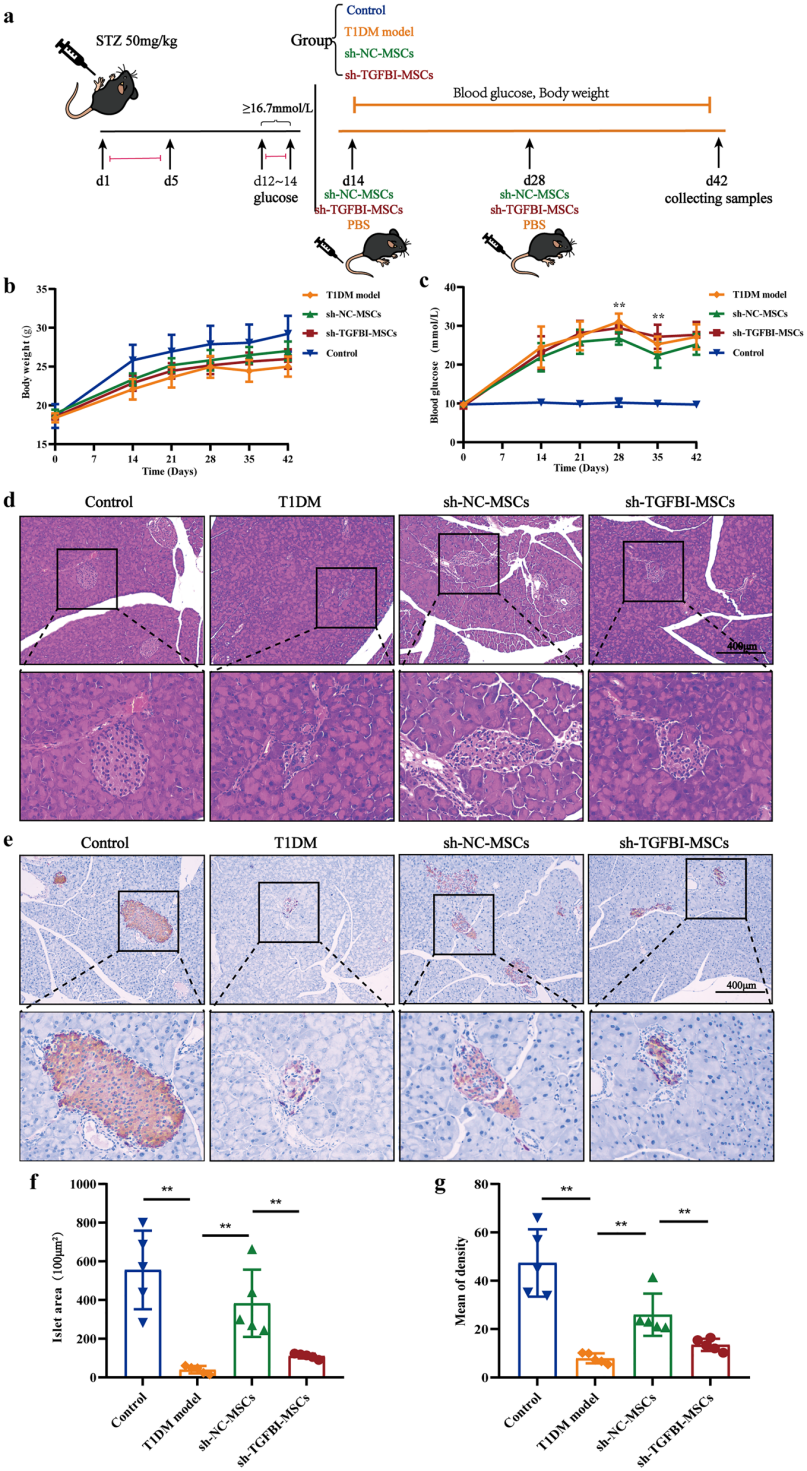
TGFBI knockdown impaired the therapeutic efficacy of hUC-MSCs in T1DM mice. **a** Experimental timeline of STZ-induced T1DM mice that were administered hUC-MSCs. **b** and **c** The weight growth curves and blood glucose are shown (*n* = 6, three independent experiments). **d** and **e** Pathological changes in pancreatic tissue were assessed by H&E staining and immunohistochemical staining after sh-TGFBI-MSCs infusion for 30 days. **f** and **g** Quantification of the islet area of the pancreas and the mean density of insulin-positive cells. *n* = 5, three independent experiments were performed, **P < 0.01

To evaluate the role of TGFBI in the treatment effects of hUC-MSCs in T1DM mice, sh-TGFBI-MSCs and sh-NC-MSCs were intravenously administered to STZ-induced T1DM mice (Fig. 2a). As shown in Fig. 2b, the average body weight of the sh-TGFBI-MSCs group was lower than that of the sh-NC-MSCs group, but the blood glucose levels were higher than those of the sh-NC-MSCs group (Fig. 2c).

We further examined the pathological changes in sh-TGFBI-MSC-treated T1DM mice, and histological examination of pancreatic tissues was performed by H&E staining and immunohistochemical staining after sh-TGFBI-MSCs infusion for 30 days. As shown in Fig. 2d and f, the islet areas in the sh-TGFBI-MSCs group were smaller than those in the sh-NC-MSCs group, indicating that TGFBI knockdown hUC-MSCs failed to preserve the destructive islets in T1DM. Moreover, the area and the mean density of insulin in the pancreas of sh-TGFBI-MSC-treated mice were smaller than those in the sh-NC-MSCs group (Fig. 2e and  g). These findings indicated that the therapeutic effects of sh-TGFBI-MSCs were weaker than those of sh-NC-MSCs in T1DM mice.

### Knockdown of TGFBI weakened the immunosuppressive capacity of hUC-MSCs in T1DM mice

**Fig. 3:**
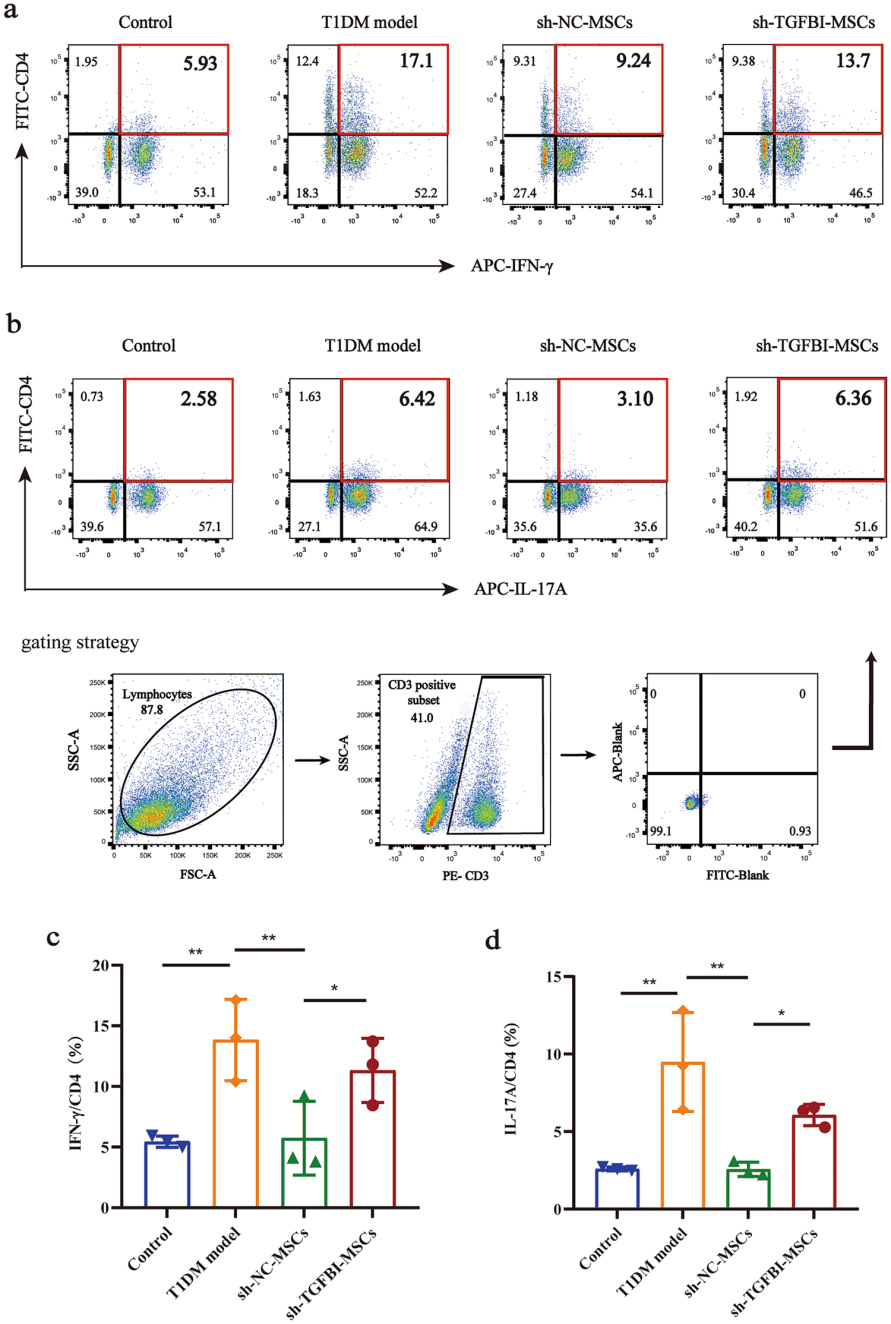
TGFBI knockdown hUC-MSCs enhanced the expression of proinflammatory cytokines in T1DM mice. **a** The proportion of CD3^+^CD4^+^IFN-γ^+^ cells was analyzed by flow cytometry. **c** Comparison of the proportion of CD3^+^CD4^+^IFN-γ^+^ cells in each group. **b** and **d** The proportion of CD3^+^CD4^+^IL-17A^+^ and quantitative results. Three independent experiments were performed, **P < 0.01,*P＜0.05

Type 1 diabetes (T1DM) is caused by autoimmune destruction resulting in pancreatic β-cells mediated by T cells. Th1 cells and Th17 cells are considered to play pivotal roles in the pathogenesis of T1DM [25, 26]. To investigate whether TGFBI affects the immunosuppressive capacity of hUC-MSCs in T1DM mice, we analyzed the expression of the proinflammatory cytokines IFN-γ and IL-17A in spleen lymphocytes using flow cytometry. The results showed that the proportion of Th1 cells (CD3^+^CD4^+^IFN-γ^+^ T cells) in the T1DM group was significantly higher than that in the other groups. However, the proportion of Th1 cells in sh-TGFBI-MSC-treated mice was higher than that in sh-NC-MSC-treated mice (Fig. 3a, c, **P  <  0.01,*P  <  0.05). Moreover, the level of IL-17A in sh-TGFBI-MSC-treated mice was lower than that in T1DM mice but higher than that in sh-NC-MSC-treated mice (Fig. 3b, d, **P < 0.01, *P <  0.05). These data suggest that knockdown of TGFBI weakens the immunosuppressive capacity of hUC-MSCs in T1DM mice.

### Knockdown of TGFBI lessened hUC-MSCs repressing T-cell proliferation

**Fig. 4:**
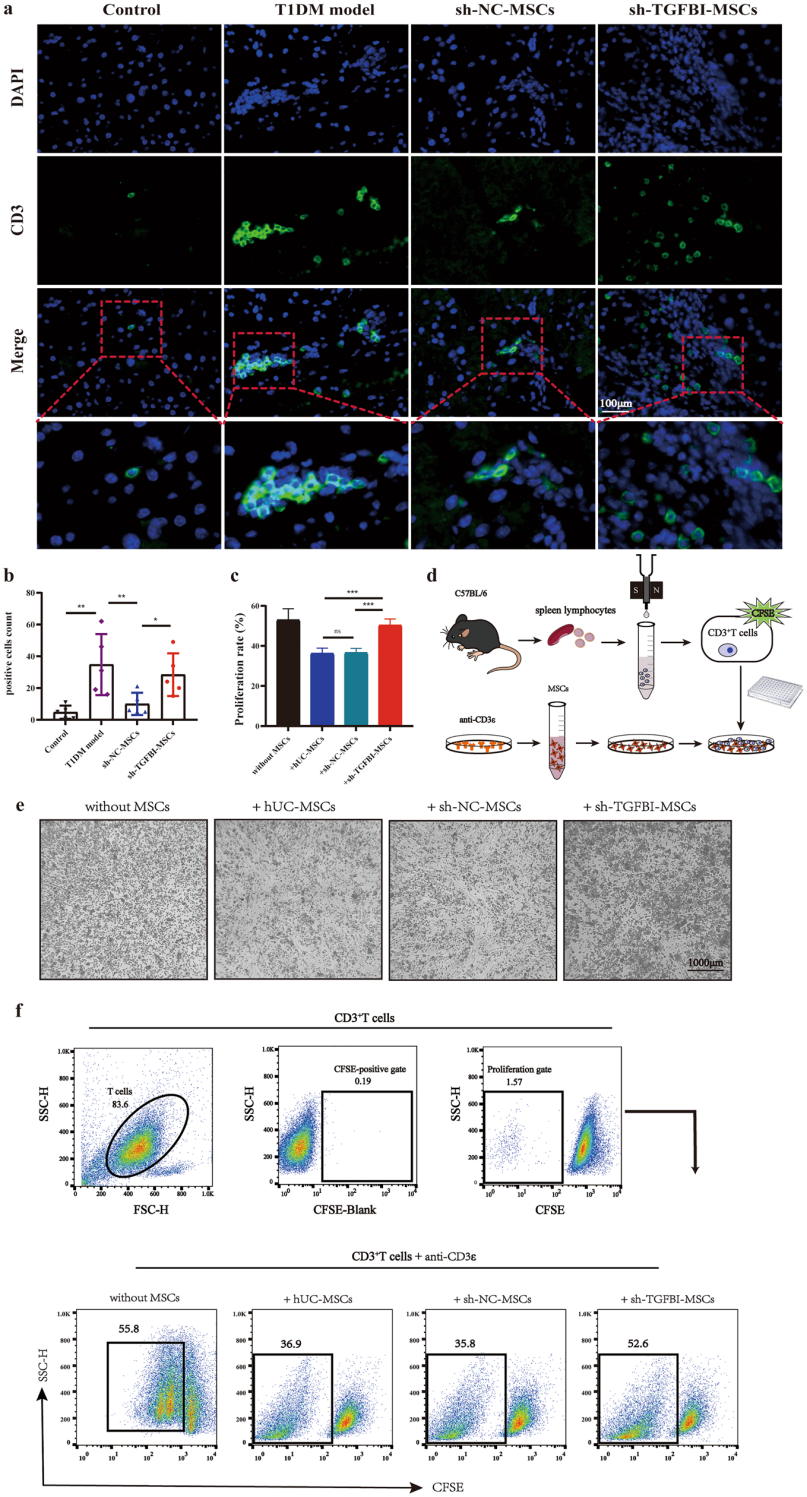
Knockdown of TGFBI lessened hUC-MSCs and repressed T-cell proliferation. **a** CD3^+^ T cells in the pancreas were detected by immunofluorescent staining. **b** Comparison of the number of CD3^+^ T cells in various groups. **d** Outline of the protocol used for the coculture assay. **e** The proliferation effects of CD3^+^ T cells in various groups were observed by microscopy. **f** A flow cytometry assay was used to analyze the proliferative effect of CD3^+^ T cells labeled with CFSE in various groups. **c** The quantitative results of the proliferation of CD3^+^ T cells in the sh-TGFBI-MSCs group were higher than those in the sh-NC-MSCs group and hUC-MSCs group. Three independent experiments, *P < 0.05, **P < 0.01, ***P < 0.001

To further evaluate whether TGFBI impaired the immunosuppressive capacity of hUC-MSCs in the pancreatic microenvironment of T1DM mice, we analyzed the infiltration of T-cell proliferation in the pancreas by immunofluorescence analysis in T1DM mice after MSCs treatment for 7 days. We labeled T cells with green fluorescent-labeled CD3 antibody and then counted the number of green-labeled cells. As shown in Fig. 4a and b, the number of green-labeled CD3^+^ T cells in the sh-TGFBI-MSCs group was significantly higher than that in the sh-NC-MSCs group, but it was lower than that in the T1DM group.

Additionally, in vitro T-cell proliferation experiments were performed to explore the role of TGFBI in the immunosuppression ability of hUC-MSCs. We conducted a coculture assay of hUC-MSCs with CD3^+^ T cells (Fig. 4d). CD3^+^ T cells were sorted by CD3 MicroBeads and labeled with CFSE. After coculture for 3 days, CD3^+^ T cells were collected and analyzed by flow cytometry. We found that hUC-MSCs and sh-NC-MSCs could strongly suppress the proliferation of activated T cells, while these suppressive capacities were obviously diminished in the sh-TGFBI-MSCs group, indicating that TGFBI knockdown could reduce the immunosuppressive capacity of hUC-MSCs (Fig. 4c, e and f). These findings reveal an important role of TGFBI in inhibiting activated T cells of hUC-MSCs in vitro and in vivo.

### hUC-MSCs secreted TGFBI inhibit T-cell proliferation by downregulating CyclinD2 expression

**Fig. 5:**
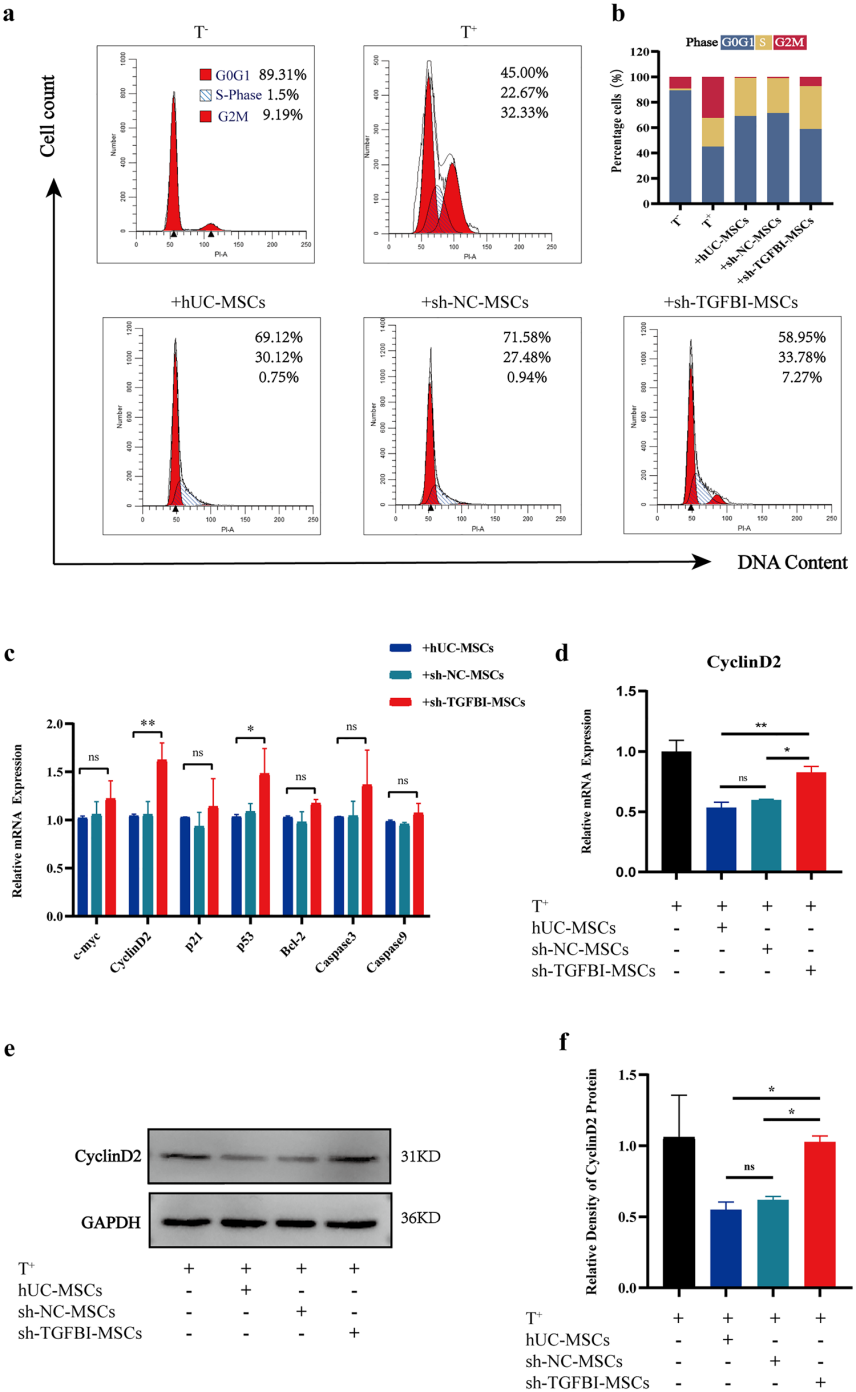
HUC-MSC-derived TGFBI inhibits T-cell proliferation by downregulating CyclinD2 expression. **a** and **b** The cell cycle in various groups was analyzed by flow cytometry, and quantitative results of the cell cycle are presented. **c** qPCR was used to analyze the expression of c-myc, CyclinD2, p21, p53, Bcl-2, Caspase 3 and Caspase 9. **d** The mRNA expression of CyclinD2 was detected by qPCR. **e** and **f** The protein expression of CyclinD2 was detected by Western blot in the above groups, and quantitative analysis is presented. The data are representative of three independent experiments. *P < 0.05, **P < 0.01, ***P < 0.001

Studies have demonstrated that one of the important mechanisms by which MSCs inhibit T-cell proliferation is by repressing T-cell cycle progression [27]. To investigate whether TGFBI could affect T-cell cycle development, we measured the change in the T-cell cycle in a hUC-MSCs and T-cell coculture system. As shown in Fig. 5a and b, the percentage of G_2_/M phase cells in the sh-TGFBI-MSCs group was markedly increased compared to that in the sh-NC-MSCs group and hUC-MSCs group. This result indicated that TGFBI should be related to T-cell cycle progression. Moreover, previous studies showed that cyclins D2 and D3 were predominantly expressed in T cells, which were associated with DNA replication and induced T-cell proliferation [28, 29]. Thus, we further detected the expression of several genes associated with the proliferation and apoptosis of T cells, including c-myc, CyclinD2, p21, p53, Bcl-2, Caspase3, and Caspase9. We found that the expression of CyclinD2 was significantly increased in the sh-TGFBI-MSCs group (Fig. 5c and d). Consistent with the mRNA expression levels, Western blot assays showed that the sh-TGFBI-MSCs group had a higher level of CyclinD2 than the sh-NC-MSCs and hUC-MSCs groups (Fig. 5e and f). Thus, our data suggest that hUC-MSC-secreted TGFBI represses T-cell proliferation by inhibiting CyclinD2 expression.

## Discussion

In the current study, we found that the TGFBI gene is essential for hUC-MSC immunologic function. hUC-MSCs with low TGFBI expression show decreased immunosuppressive capability and display impaired therapeutic effects in T1DM mice. hUC-MSC knockdown of TGFBI markedly increased pathological lesions in a mouse model of T1DM. A higher number of infiltrating T cells was detected in the pancreas of T1DM mice. Moreover, the expression of IFN-γ and IL-17A was increased in the spleen. hUC-MSC-derived TGFBI repressed activated T-cell proliferation by interfering with CyclinD2 expression.

MSCs are multipotent progenitor cells that can inhibit the proliferation and function of T and B cells and NK cells [30]. MSC-mediated immunosuppression is also associated with the inhibition of proinflammatory cytokine induction [31]. The results of in vivo administration of MSCs in autoimmune diseases are consistent with in vitro studies. T1DM, which was previously thought to be a T-cell-mediated autoimmune disease, is now treated by MSCs, which have been considered a promising treatment modality for T1DM in recent years [32, 33]. We and others have previously demonstrated the benefit of MSC therapy in the specific setting of T1DM [9, 34]. Importantly, our previous work demonstrated the preventive effect of MSCs in vivo using an STZ-induced T1DM mouse model [35]. However, although the therapeutic value of MSCs for attenuating autoimmune disorders, including T1DM, has potential, MSCs treatment of this disease remains largely unexplored, especially the mechanism of MSCs immune regulation.

In this report, we first demonstrate that TGFBI is an important gene in regulating MSC immunomodulatory function in a T1DM mouse model. We used RNA sequencing to identify that TGFBI was highly expressed in hUC-MSCs. TGFBI is one of the downstream genes of TGF-β signaling [36]. Previous evidence has demonstrated that TGF-β derived from MSCs can inhibit lymphocyte proliferation and mediate the differentiation of regulatory T cells [37]. TGFBI is an extracellular matrix protein that plays a vital role in promoting islet survival and function and inhibiting the activation of T-cell activation [18, 19]. Thus, we speculate that TGFBI may be involved in the regulation of the immunoregulatory effect of hUC-MSCs. To investigate the role of TGFBI in the immunologic function of hUC-MSCs, we constructed a lentivirus vector to knock down TGFBI in hUC-MSCs (sh-TGFBI-MSCs) and control cells (sh-NC-MSCs). We found that sh-TGFBI-MSCs retained the properties of MSCs in vitro. Furthermore, we established an STZ-induced T1DM mouse model, and then sh-TGFBI-MSCs were transplanted into T1DM mice. We found that the therapeutic effects, including weight recovery, blood glucose levels, and improved pancreatic damage, were lower in mice that received sh-TGFBI-MSCs infusion than in the sh-NC-MSC group mice. Our data revealed that the immunosuppressive effects of MSCs on T1DM were more likely to depend on TGFBI.

It has been reported that hUC-MSCs cure T1DM by suppressing T lymphocyte activation and proliferation [38, 39], and our data showed that hUC-MSCs suppress T lymphocyte proliferation in pancreatic tissue. These results are consistent with observations reported by others. A previous study demonstrated that T cells are the key mediators of the adaptive immune system in animal and human tissues [40, 41]. Th1 and Th17 cells differentiated by CD4^+^ T lymphocytes could produce proinflammatory cytokines that prolong the autoimmune condition, leading to a failure of immune tolerance to β-cells in T1DM [42]. Our data indicated that compared to sh-NC-MSC transplantation, sh-TGFBI-MSC transplantation could significantly upregulate the abundance of Th1 and Th17 cells in T1DM mice and promote the expression of IFN-γ and IL-17A in T cells. These results suggest that TGFBI is involved in the immunoregulation of MSCs. However, little is known about the molecular mechanism by which MSCs suppress T lymphocyte proliferation by TGFBI.

It should be noted that MSCs prevent T lymphocyte proliferation by preventing cells from entering G_0_/G_1_ to enter S phase [27, 43]. We examined whether the cell cycle of activated T lymphocytes was affected by TGFBI derived from hUC-MSCs. In the presence of sh-TGFBI-MSCs, the number of T lymphocytes in G_2_/M phase was increased. In general, T-cell proliferation is dependent on progression through the cell cycle through the G_1_/S and G_2_/M checkpoints. This progression is regulated by cyclin protein [44, 45]. Hence, we speculated that the immunosuppressive effects of TGFBI on T cells were due to inhibiting cyclin protein. This hypothesis was further validated at the mRNA and protein levels. Our data demonstrated that hUC-MSC-derived TGFBI indeed decreased CyclinD2 expression in T cells. In contrast, knocking down TGFBI in hUC-MSCs abrogated the inhibition of CyclinD2 expression in T cells. However, several studies have demonstrated that mesenchymal stem/stromal cell-secreted TGFB could regulate Treg production to relieve inflammation. Interestingly, TGFBI also belongs to the TGFB family, which may affect T-cell function through another pathway [46, 47]. The complete mechanism by which TGFBI regulates the immunosuppressive capacity of hUC-MSCs still needs further investigation.

Based on the evidence presented above, we conclude that TGFBI secreted from hUC-MSCs could suppress the proliferation of activated T cells and thus ameliorate STZ-induced T1DM in a mouse model. Mechanistic analysis showed that hUC-MSCs could function through TGFBI/CyclinD2 to regulate effector T cells. Our findings serve as evidence supporting that TGFBI plays an important role in regulating the immunomodulatory function of hUC-MSCs. TGFBI derived from hUC-MSCs repressed activated T-cell proliferation by interfering with CyclinD2 expression.

## Conclusions

This study demonstrated a new role for TGFBI in regulating the immunomodulatory capability of hUC-MSCs and the therapeutic effect of T1DM. We found that TGFBI secreted from hUC-MSCs could inhibit T-cell proliferation by repressing CyclinD2 expression. On this basis, we concluded that hUC-MSC-secreted TGFBI could alleviate STZ-induced T1DM by inhibiting CyclinD2 expression in T-cell proliferation.

## Supplementary Information

Below is the link to the electronic supplementary material.

Supplementary file1 Supplementary Figure 1. Identification of hUC-MSCs transduced with shRNA for a nontargeting sequence (sh-NC). a. Heat map of differential gene expression in various MSCs, including BM-MSCs, hP-MSCs, hM-MSCs, and hUC-MSCs. b. TGFBI expression in different MSCs shown by qPCR. c. Flow cytometry analysis was used to detect the cell surface markers of sh-NC-MSCs. d. EGFP expression in sh-NC-MSCs was observed by fluorescence microscopy. e. The adherent cells (P3) were stained with Wright-Giemsa. f and g. Adipogenic differentiation of sh-NC-MSCs was detected by Oil red O staining. j. qPCR analysis of PPAR-γ and adipsin mRNA expression in the adipogenic induction group. h and i. Osteogenic differentiation of sh-TGFBI-MSCs was shown by alkaline phosphatase (ALP) staining. k. qPCR analysis of ALP and osteopontin (OPN) mRNA expression in the osteogenic induction group. l and m. Chondrogenic induction of sh-NC-MSCs was shown by Alcian Blue staining. n. hUC-MSCs were successively sub-cultured from P3 to P10, and the expression of TGFBI and TGF-β1 in different passage of hUC-MSCs was detected by qPCR. o. hUC-MSCs were treated with IFN-γ (20 ng/ml) and TNF-α (20 ng/ml) for 24 h, TGFBI and TGF-β1 mRNA levels were measured by qPCR. The data are shown from one representative experiment of three replicates. ***P< 0.001 (TIF 34989 KB)

Supplementary file2 (TIF 361 KB)

Supplementary file3 (TIF 361 KB)

Supplementary file4 (TIF 642 KB)

Supplementary file5 (TIF 361 KB)

Supplementary file6 (DOCX 12 KB)

## Data Availability

All data generated or analyzed during this study are included in this published article and its supplementary information files.
