# Supplementary figures and images for: Human umbilical cord mesenchymal stem cell-derived TGFBI attenuates streptozotocin-induced type 1 diabetes mellitus by inhibiting T-cell proliferation
Source: Hum Cell. 2023 Feb 25;36(3):997–1010. doi: 10.1007/s13577-023-00868-9 (PMC10110644; doi:10.1007/s13577-023-00868-9)

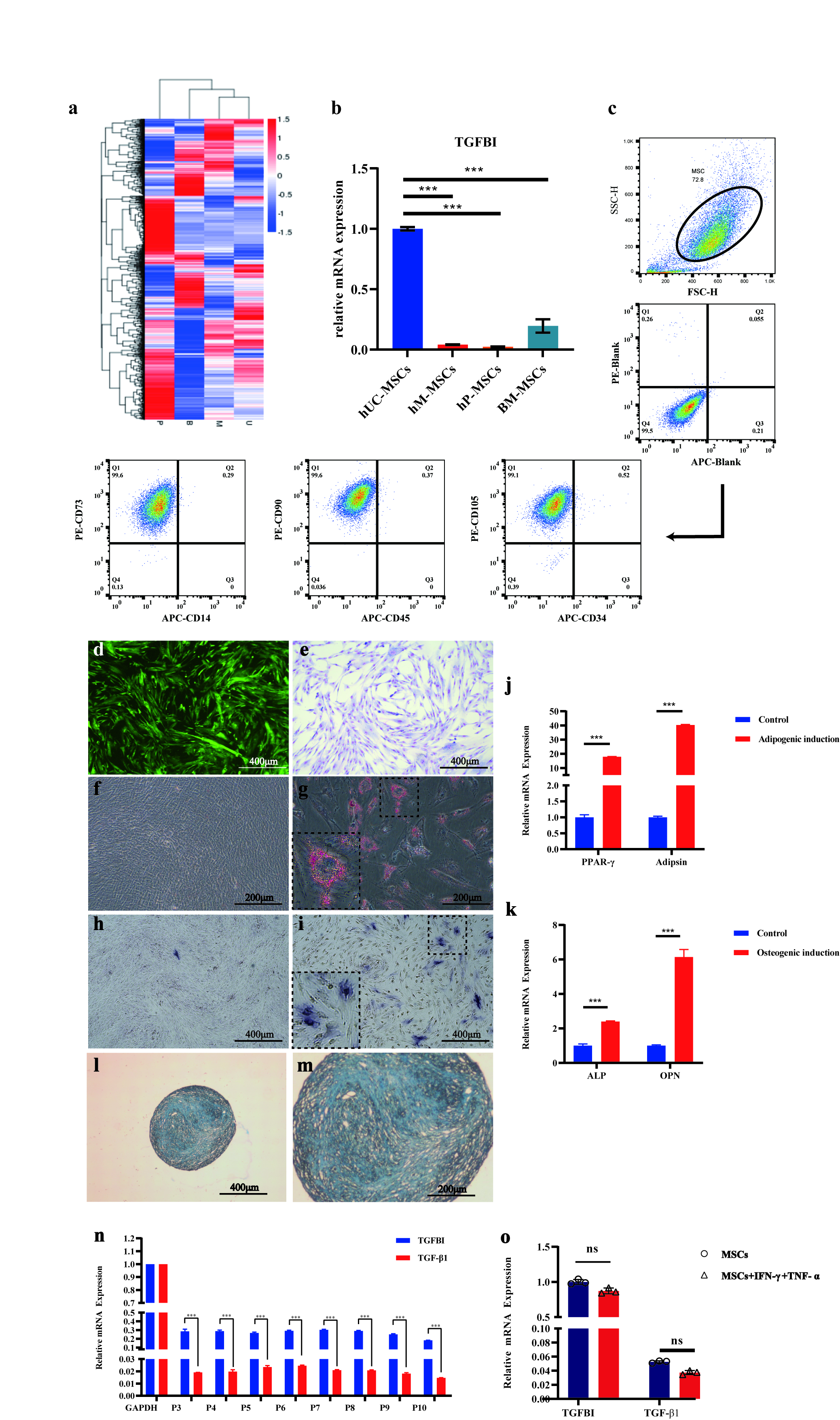

Supplement: Supplementary file 1 — Supplementary file1 Supplementary Figure 1. Identification of hUC-MSCs transduced with shRNA for a nontargeting sequence (sh-NC). a. Heat map of differential gene expression in various MSCs, including BM-MSCs, hP-MSCs, hM-MSCs, and hUC-MSCs. b. TGFBI expression in different MSCs shown by qPCR. c. Flow cytometry analysis was used to detect the cell surface markers of sh-NC-MSCs. d. EGFP expression in sh-NC-MSCs was observed by fluorescence microscopy. e. The adherent cells (P3) were stained with Wright-Giemsa. f and g. Adipogenic differentiation of sh-NC-MSCs was detected by Oil red O staining. j. qPCR analysis of PPAR-γ and adipsin mRNA expression in the adipogenic induction group. h and i. Osteogenic differentiation of sh-TGFBI-MSCs was shown by alkaline phosphatase (ALP) staining. k. qPCR analysis of ALP and osteopontin (OPN) mRNA expression in the osteogenic induction group. l and m. Chondrogenic induction of sh-NC-MSCs was shown by Alcian Blue staining. n. hUC-MSCs were successively sub-cultured from P3 to P10, and the expression of TGFBI and TGF-β1 in different passage of hUC-MSCs was detected by qPCR. o. hUC-MSCs were treated with IFN-γ (20 ng/ml) and TNF-α (20 ng/ml) for 24 h, TGFBI and TGF-β1 mRNA levels were measured by qPCR. The data are shown from one representative experiment of three replicates. ***P< 0.001 (TIF 34989 KB) [file 13577_2023_868_MOESM1_ESM.tif]

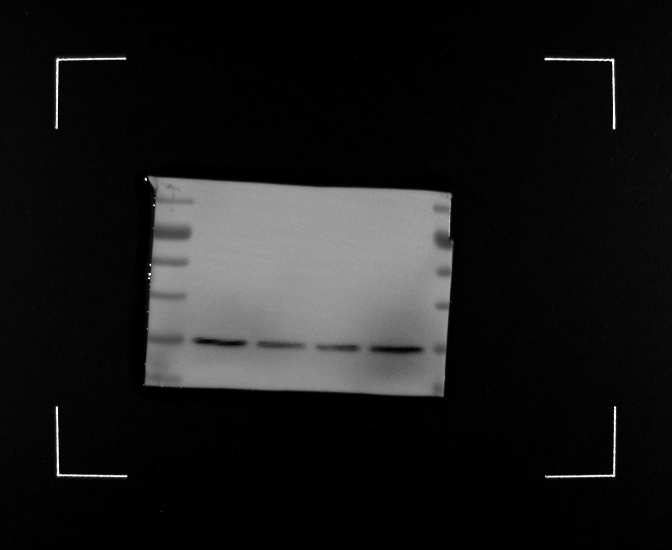

Supplement: Supplementary file 2 — Supplementary file2 (TIF 361 KB) [file 13577_2023_868_MOESM2_ESM.tif]

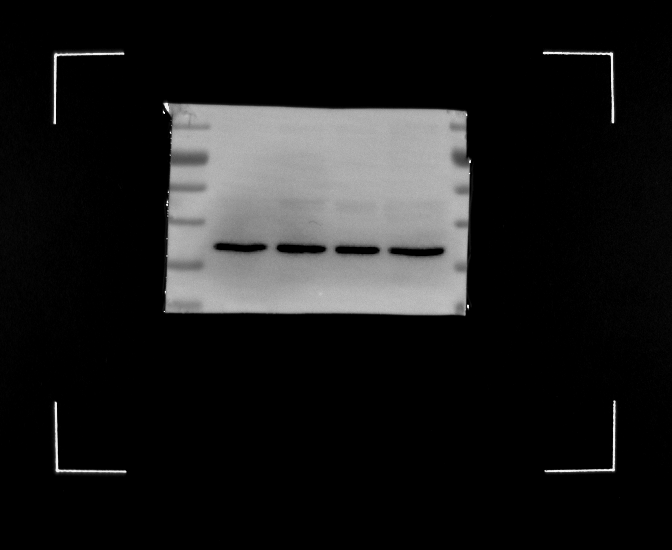

Supplement: Supplementary file 3 — Supplementary file3 (TIF 361 KB) [file 13577_2023_868_MOESM3_ESM.tif]

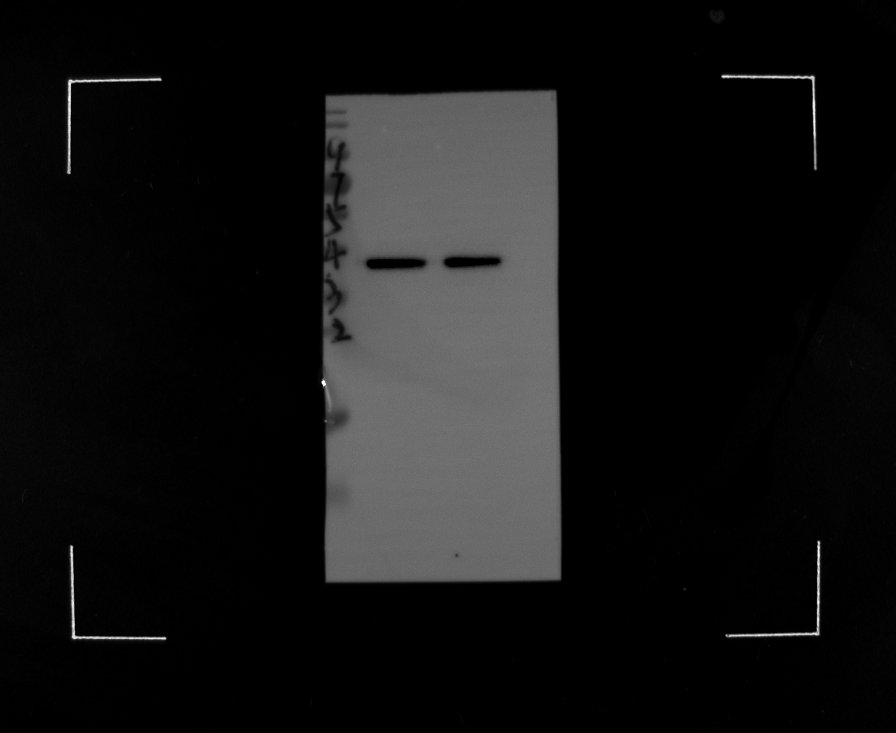

Supplement: Supplementary file 4 — Supplementary file4 (TIF 642 KB) [file 13577_2023_868_MOESM4_ESM.tif]

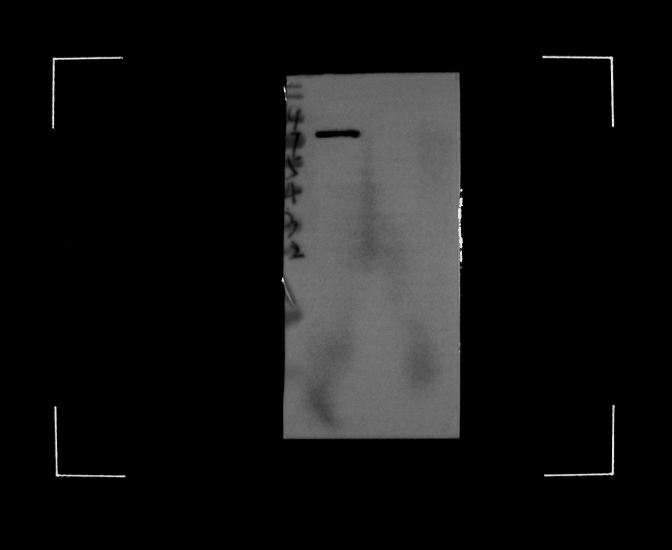

Supplement: Supplementary file 5 — Supplementary file5 (TIF 361 KB) [file 13577_2023_868_MOESM5_ESM.tif]
