# Supplementary material for: Human umbilical cord mesenchymal stem cell-derived TGFBI attenuates streptozotocin-induced type 1 diabetes mellitus by inhibiting T-cell proliferation
Source: Hum Cell. 2023 Feb 25;36(3):997–1010. doi: 10.1007/s13577-023-00868-9 (PMC10110644; doi:10.1007/s13577-023-00868-9)
Supplement: Supplementary file 6 — Supplementary file6 (DOCX 12 KB) [file 13577_2023_868_MOESM6_ESM.docx]

| ID | Forward 5’-3’ | Reverse 5’-3 |
| --- | --- | --- |
| Homo GAPDH | TCAAGATCATCAGCAATGCC | CGATACCAAAGTTGTCATGGA |
| Homo TGFBI | CAGAAGGTTATTGGCACTAATAGG | CTGATGACTGTTGATTTGCCA |
| Homo PPAR-γ | GACCACTCCCACTCCTTTGA | ATTCAATTGCCATGAGGGAG |
| Homo Adipsin | TCACCCAAGCAACAAAGTC | AAAGACCAACCAGATGCAG |
| Homo ALP | CATCCTGTATGGCAATGGG | TGTTGTGAGCATAGTCCAC |
| Homo OPN | CCATACCAGTTAAACAGGCTG | TCAGGGTTTAGCCATGTGG |
| Mus GAPDH | ACTCTTCCACCTTCGATGC | CCGTATTCATTGTCATACCAGG |
| Mus CyclinD2 | TCCTATTTCAAGTGCGTGC | CTCACAGACCTCTAGCATCC |

Table 1：Primer sequence
